# Supplementary material for: Crystal Structure-Guided Design of Bisubstrate Inhibitors and Photoluminescent Probes for Protein Kinases of the PIM Family
Source: Molecules. 2021 Jul 19;26(14):4353. doi: 10.3390/molecules26144353 (PMC8307404; doi:10.3390/molecules26144353)
Supplement: Supplementary file 1 [file molecules-26-04353-s001.zip › molecules-1265068_Supplementary data.pdf]

Crystal Structure-Guided Design of Bisubstrate Inhibitors and Photoluminescent Probes for Protein Kinases of the PIM Family

Olivier E. Nonga [1], Darja Lavogina [1], Erki Enkvist [1], Katrin Kestav [1], Apirat Chaikuad [2,3], Sarah E. Dixon-Clarke [4,5], Alex N. Bullock [4], Sergei Kopanchuk [1], Taavi Ivan [1], Ramesh Ekambaram [1], Kaido Viht [1], Stefan Knapp [2,3] and Asko Uri [1,*]

[1] Institute of Chemistry, University of Tartu, 14A Ravila St., 50411 Tartu, Estonia; olivier.etebe.nonga@ut.ee (O.E.N.); darja.lavogina@ut.ee (D.L.); erki.enkvist@ut.ee (E.E.); katrin.kestav@ut.ee (K.K.); sergei.kopanchuk@ut.ee (S.K.); taavi.ivan@ut.ee (T.I.); ramcheme@gmail.com (R.E.); Kaido.viht@ut.ee (K.V.)

[2] Institut für Pharmazeutische Chemie, Goethe University Frankfurt am Main, Max-von-Laue-Str. 9, 60438 Frankfurt am Main, Germany; chaikuad@pharmchem.uni-frankfurt.de (A.C.); knapp@pharmchem.uni-frankfurt.de (S.K.)

[3] Structural Genomics Consortium (SGC), Buchmann Institute for Life Sciences, Goethe University Frankfurt, Max-von-Laue-Str. 15, 60438 Frankfurt am Main, Germany

[4] Centre for Medicines Discovery, University of Oxford, Old Road Campus, Roosevelt Drive, Oxford OX3 7DQ, UK; alex.bullock@cmd.ox.ac.uk

[5] Department of Cancer Biology, Dana-Farber Cancer Institute, Longwood Center, 450 Brookline Avenue, Boston, MA 02215, USA; sarah.dixon-clarke@dfci.harvard.edu

* Correspondence: asko.uri@ut.ee; Tel.: +372-737-5275

Supplementary Materials:

Supplementary Methods ... 3

1. Synthesis of methyl 3-amino-6-bromothieno[3,2-*b*]pyridine-2-carboxylate (**B**) ... 3

2. Synthesis of 7-bromo-2-(chloromethyl)pyrido[4,5]thieno[3,2-*d*]pyrimidin-4-one (**D**) ... 3

3. General protocol of solid phase synthesis ... 3

Supplementary References ... 5

Supplementary Tables ... 6

Table S1. Data collection and refinement statistics of PIM-1/ARC co-crystals ... 6

Table S2. Structural formulae and HRMS data of the novel compounds in the study ... 7

Table S3. Selectivity profiling of ARC inhibitors (1 μM) towards a panel of PKs ... 14

Table S4. HPLC (C18 reversed phase) purity data for the previously unpublished compounds ... 19

Supplementary Figures ... 20

Figure S1. $2F_o$-F_c electron density map contoured at 1σ for the inhibitors in co-crystal structures with PIM-1 ... 20

Figure S2. Specificity of D1D2 antibody to PIM-2 detection ... 21

Figure S3. Stability of ARC-1451/PIM-2 complex in PC-3 cell lysate. ... 22

Figure S4. Time- and concentration-dependence of the cellular uptake of compound **6** into live U2OS cells. ... 23

Figure S5. Comparison of cellular uptake of compound **8** (labelled with TAMRA) and compound **6** (labelled with Cy5) into live U2OS cells. 24

Figure S6. Intracellular localization of compounds labelled with Cy5 and incorporating different number of Arg residues. 26

Figure S7. Biochemical characterization of the synthesized compounds and commercially available inhibitors....................28

Supplementary Video 30

Video S1. Colocalization studies of PIM-targeting compound **6** with fluorescently tagged PIM-1 *vs* peroxisomal marker (following 48 h transfection) in live U2OS cells....................30

Supplementary Methods

*1. Synthesis of methyl 3-amino-6-bromothieno[3,2-b]pyridine-2-carboxylate (**B**)*

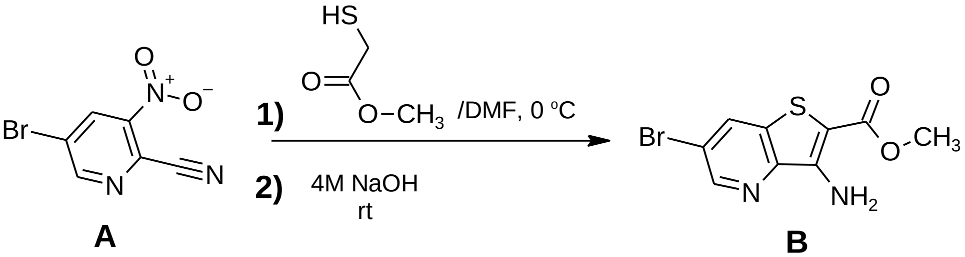

To a cold solution of 5-bromo-3-nitropyridine-2-carbonitrile (**A**, 100 mg, 569 μmol) in DMF, methyl 2-mercaptoacetate (47 μL, 569 μmol) was added dropwise at 0 °C. The reaction mixture was stirred at 0 °C for 30 min, and then 4M NaOH aqueous solution (150 μL) was added dropwise. After stirring at RT for 3 h, the reaction mixture was quenched with ice-water. The resulting precipitate was collected by filtration, washed with ethanol, and dried to yield 70 mg of yellowish solid (yield 43%). The product emitted blue fluorescence as a normal-phase thin-layer chromatography (TLC) spot with R_f = 0.5 (EtOAc/hexane 1:3). UV_{max} at 288 nm and at 374 nm (DMF). MS (ESI) m/z 288 $(M + H)^+$; ^1H NMR (700 MHz, DMSO-d_6) δ ppm 3.83 (s, 3H), 6.95 (s, 2H), 8.77 (m, 2H).

*2. Synthesis of 7-bromo-2-(chloromethyl)pyrido[4,5]thieno[3,2-d]pyrimidin-4-one (**D**)*

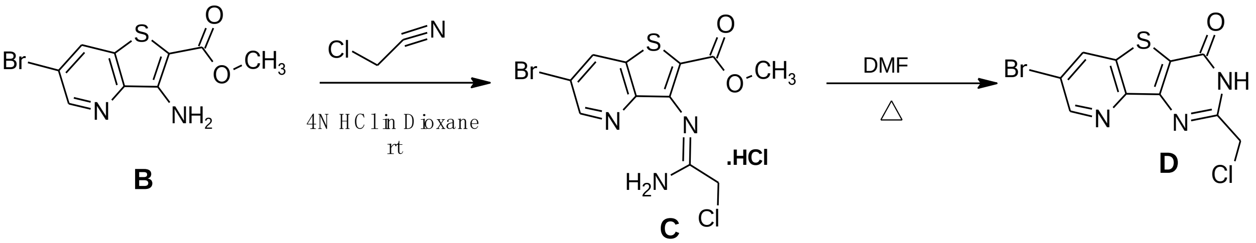

A suspension of methyl-3-amino-6-bromothieno[3,2-*b*]pyridine-2-carboxylate (**B**, 50 mg, 150 μmol) in 4 N hydrochloric acid in dioxane (2 mL) was treated with 2-chloroacetonitrile (18.5 μL, 300 μmol) at room temperature for 3 h to yield the intermediate product **C** (51 mg, yield 94%). Then, the reactional mixture was refluxed for 2 h. A white solid was collected by filtration, washed with ethanol, and dried to give the desired product (47 mg; yield 100%). The product did not emit blue fluorescence as a TLC spot with R_f = 0.5 (EtOAc/hexane 1:1). UV_{max} at 262 nm and at 312 nm (DMF). MS (ESI) m/z 332 $(M + H)^+$; ^1H NMR (700 MHz, DMSO-d_6) δ ppm: 4.69 (s, 2 H), 8.96 (s, 1H), 9.05 (s, 1H), 13.38 (bs, 1H).

3. General protocol of solid phase synthesis

The general protocol was following that in [1].

<u>3.1. ARC-3126 and compound **1**</u>

BPTP (**4**) (1.5 eq.) in DMF and DIPEA (6 eq.) was added to the N-terminal amino group of the corresponding peptide on the resin (1 eq.) and stirred slowly at 60 °C for 8 h. Then, the resin was washed, dried, and the cleavage of the product from the resin followed according to the general protocol.

3.2. Compounds **2** and **5**

A solution of 7-bromo-2-(chloromethyl)-thieno[2,3-*d*]pyrimidin-4(3*H*)-one (BBTP) (1.5 eq.) in DMF and DIPEA (6 eq.) was added to N-terminal amino group of the corresponding peptide on the resin (1 eq.) and stirred slowly at 60 °C for 8 h. Thereafter, the resin was washed, dried, and the cleavage of the product from the resin followed according to the general protocol.

3.3. Compound **3**

BBTP (3 eq.) and propargylamine (3 eq.) were dissolved in DMF in the presence of DIPEA (6 eq.) and stirred slowly at 60 °C for 18 h to obtain 8-bromo-2-[(prop-2-ynylamino)methyl]-3*H*-benzothiopheno[3,2-*d*]pyrimidin-4-one, which was used in the next step without further purification. The solution of the latter alkyne-group containing compound (1.5 eq.) in DMF was added to the azido-group containing peptide on the resin (1 eq.) in the presence of DIPEA (100 eq.) and CuI (3 eq.) and stirred slowly at RT for 24 h. Then, the resin was washed, dried, and the cleavage of the product from the resin followed according to the general protocol.

3.4. Compound **4**

BBTP (3 eq) and sodium azide (3 eq) were dissolved in DMF in the presence of DIPEA (6 eq.) and stirred slowly at 60 °C for 18 h to obtain 2-(azidomethyl)-8-bromo[1]benzothieno[3,2-*d*]pyrimidin-4(3*H*)-one, which was used in the next step without further purification. The solution of the latter azido-group containing compound (1.5 eq.) in DMF was added to the alkyne-group containing peptide on the resin (1 eq.) in the presence of DIPEA (100 eq.) and CuI (3 eq.) and stirred slowly at RT for 24 h. Then, the resin was washed, dried, and the cleavage of the product from the resin followed according to the general procedure.

3.5. (D-Arg)$_2$-Gly-D-Arg-D-Lys-NH_2 (PIM peptide)

The synthesis of this compound was performed according to the general protocol described above. No linker or adenosine analogue fragment was introduced after removal of the Fmoc group from the N-terminal D-Arg.

Supplementary References

1. Ekambaram, R.; Enkvist, E.; Vaasa, A.; Kasari, M.; Raidaru, G.; Knapp, S.; Uri, A. Selective Bisubstrate Inhibitors with Sub-Nanomolar Affinity for Protein Kinase Pim-1. *ChemMedChem* **2013**, *8*, 909–913, doi:https://doi.org/10.1002/cmdc.201300042.

Supplementary Tables

Table S1. Data collection and refinement statistics of PIM-1/ARC co-crystals

**Complex**	**PIM-1-ARC-1411**	**PIM-1-ARC-1415**	**PIM-1-ARC-3126**
PDB codes	7OOV	7OOW	7OOX
Beamline	Diamond I02	Diamond I03	Diamond I03
Data Collection[a]			
Resolution (Å)	48.98-1.96 (2.03-1.96)	42.70-1.95 (2.02-1.95)	47.4-1.97 (2.04-1.97)
Space group	*P* 6_5	*P* 6_5	*P* 6_5
Cell dimensions	a=b=98.0, c=80.8 Å<br>α=β=90.0°, γ=120.0°	a=b=98.6, c=80.7 Å<br>α=β=90.0°, γ=120.0°	a=b=94.8, c=80.4 Å<br>α=β=90.0°, γ=120.0°
Number of unique reflections	31,721 (3,086)	32,555 (3,196)	29,127 (2,853)
Completeness (%)	100.0 (100.0)	99.9 (99.9)	100.0 (100.0)
I/σI	9.4 (2.0)	11.0 (3.1)	9.4 (2.0)
R_{merge} (%)	0.059 (0.736)	0.099 (0.948)	0.120 (0.679)
CC (1/2)	0.999 (0.709)	0.995 (0.734)	0.995 (0.759)
Redundancy	5.6 (5.4)	5.1 (5.0)	5.0 (4.9)
Refinement			
Number atoms in refinement (P/L/O)[b]	2,217/ 104/ 178	2,217/ 93/ 231	2,325/ 100/ 275
B factor (P/L/O)[b] (Å^2)	45/ 72/ 51	32/ 51/ 41	27/ 49/ 35
R_{fact} (%)	16.2	16.0	17.9
R_{free} (%)	19.5	18.4	22.2
rms deviation bond[c] (Å)	0.014	0.013	0.014
rms deviation angle[c] (°)	1.4	1.4	1.5
Molprobity Ramachandran			
Favor (%)	94.65	94.65	96.09
Outlier (%)	0	0	0
Crystallization conditions	4 °C, 5.8 mg/mL PIM-1, 22% PEG3350, 0.1 M ammonium sulfate, 0.1 M tris pH 8.5	20 °C, 7.0 mg/mL PIM-1, 15% (w/v) PEG 10000, 0.2 M magnesium chloride, 0.1 M tris pH 7.9	20 °C, 7.0 mg/mL PIM-1, 1 M ammonium phosphate dibasic, 0.1 M acetate pH 4.5

[a] Values in brackets indicate high-resolution shell statistics.

[b] P/L/O indicates protein, ligand and others.

[c] rms indicates root-mean-square.

Table S2. Structural formulae and HRMS data of the new compounds in the study

Compound	Structure	Calculated monoisotopic mass, Da	Experimental deconvoluted HRMS, Da
ARC-1451	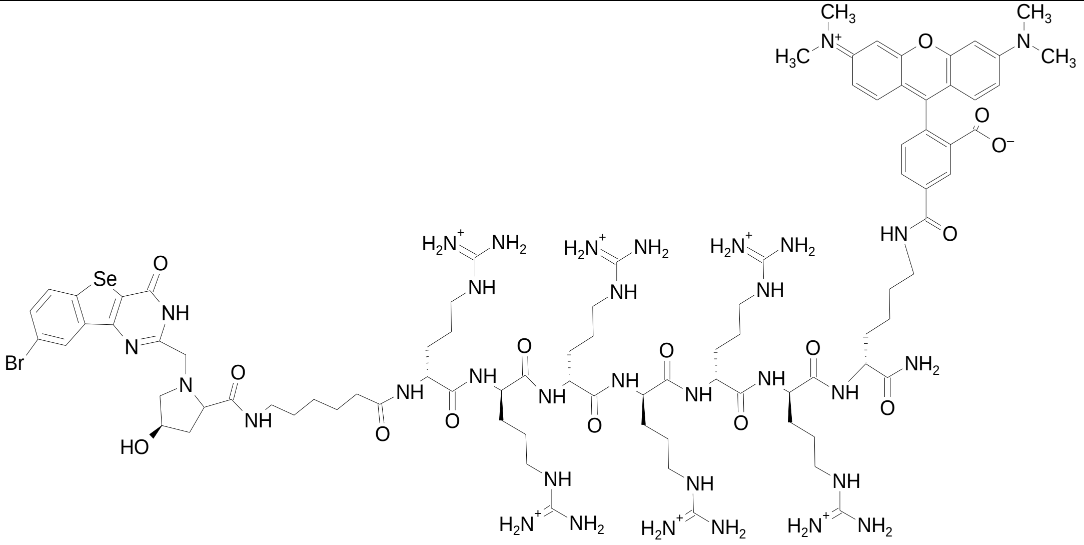 $C_{89}H_{130}BrN_{33}O_{15}Se$	2060.88455	2060.87992
ARC-3126	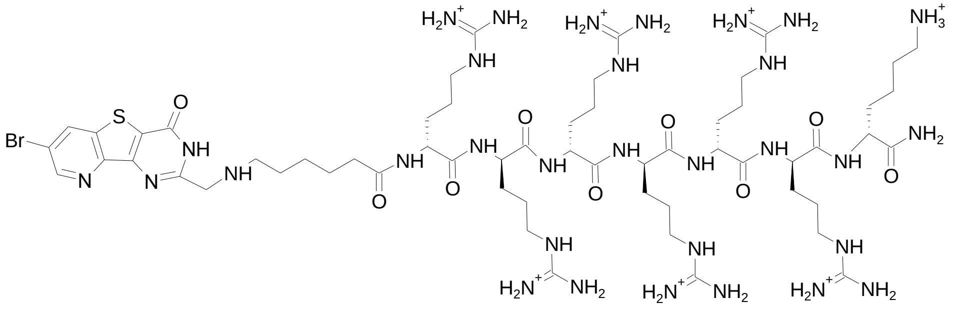 $C_{58}H_{102}BrN_{31}O_9S$	1487.7360	1487.7348

1 (ARC-2067)	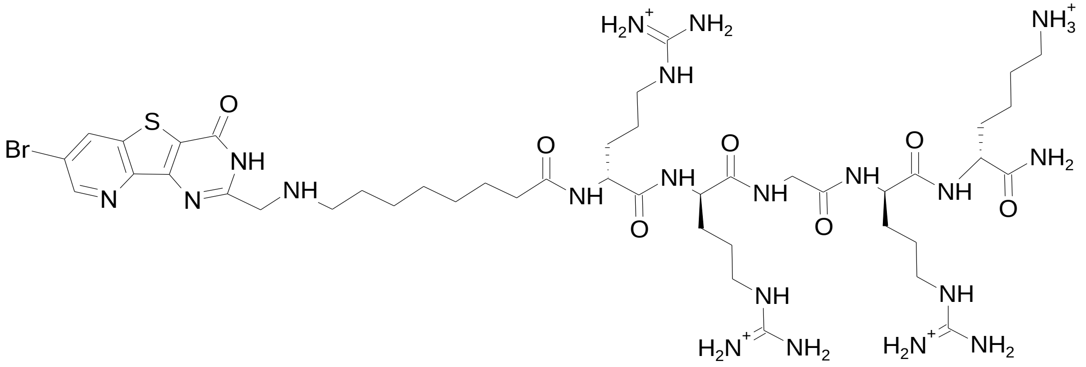 $C_{44}H_{73}BrN_{20}O_7S$	1104.48594	1104.48579
2 (ARC-2059)	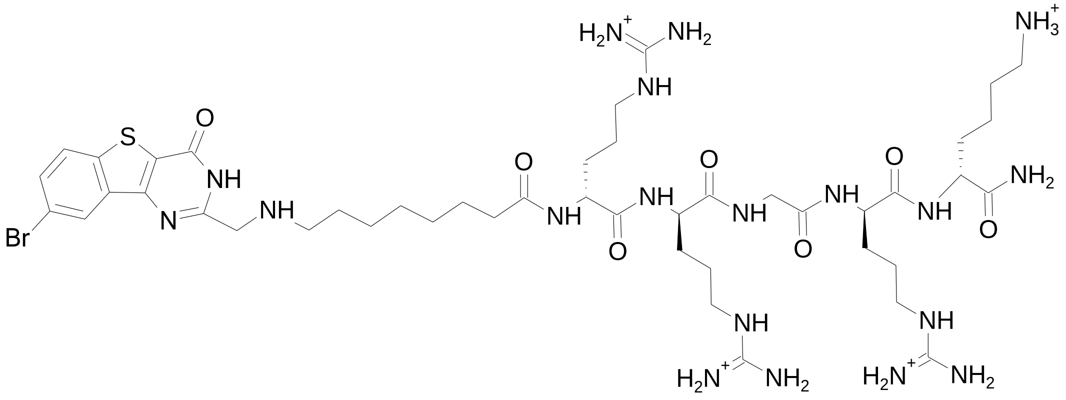 $C_{45}H_{74}BrN_{19}O_7S$	1103.49069	1103.48934

3<br>(ARC-2060)	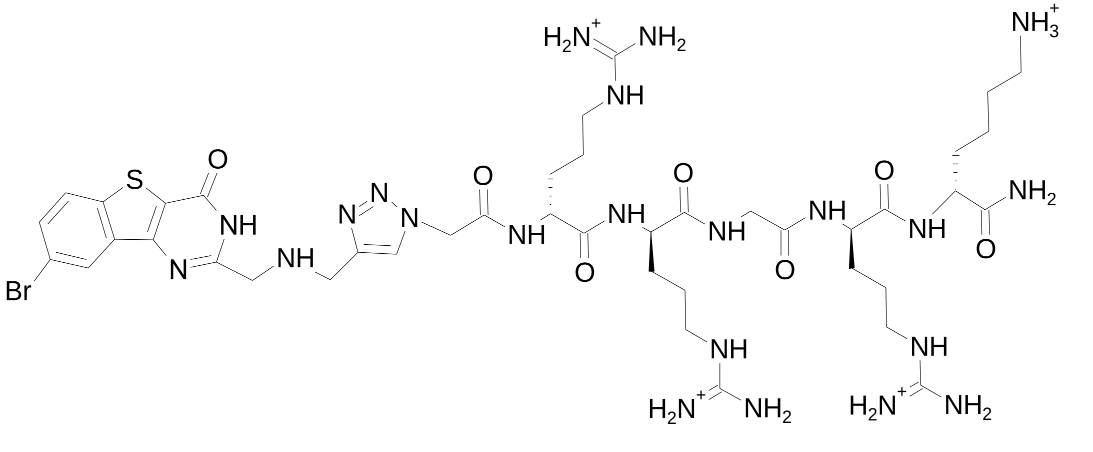<br>$C_{42}H_{65}BrN_{22}O_7S$	1100.42949	1100.42622
4<br>(ARC-2061)	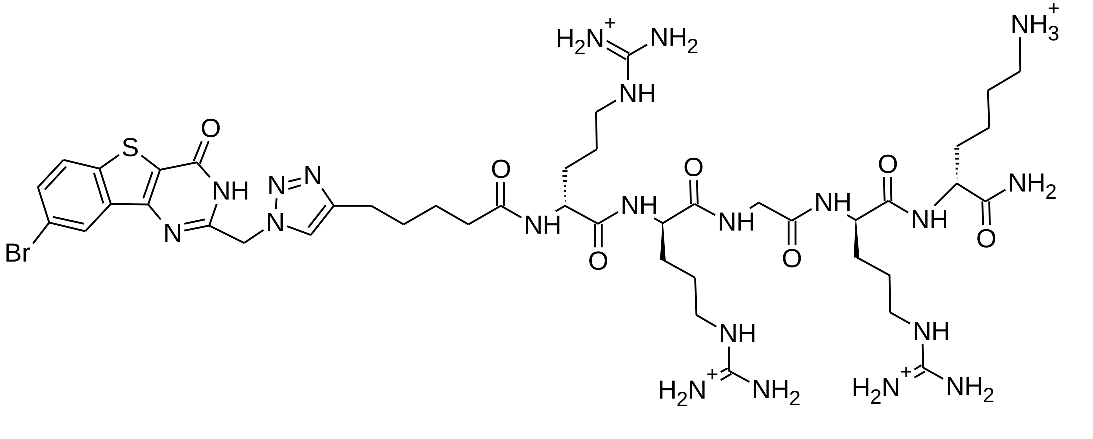<br>$C_{44}H_{68}BrN_{21}O_7S$	1113.4497	1113.4494

5<br>(ARC-2062)	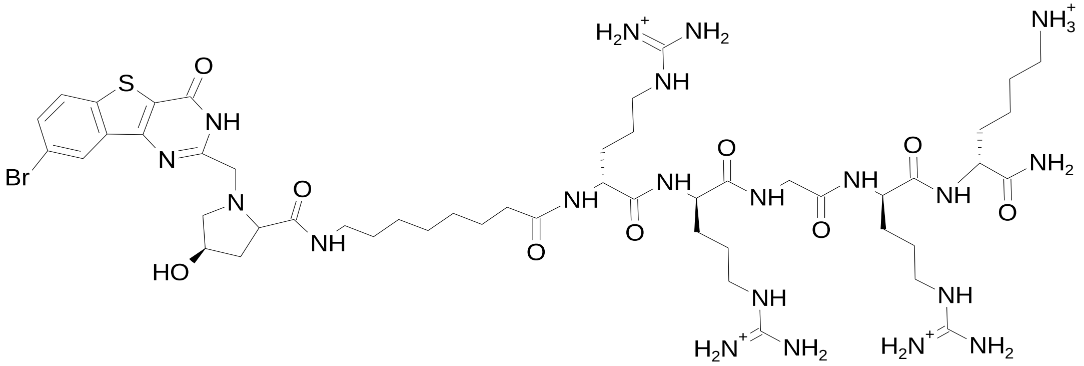<br>$C_{50}H_{81}BrN_{20}O_9S$	1218.53634	1218.61533
6<br>(ARC-2074)	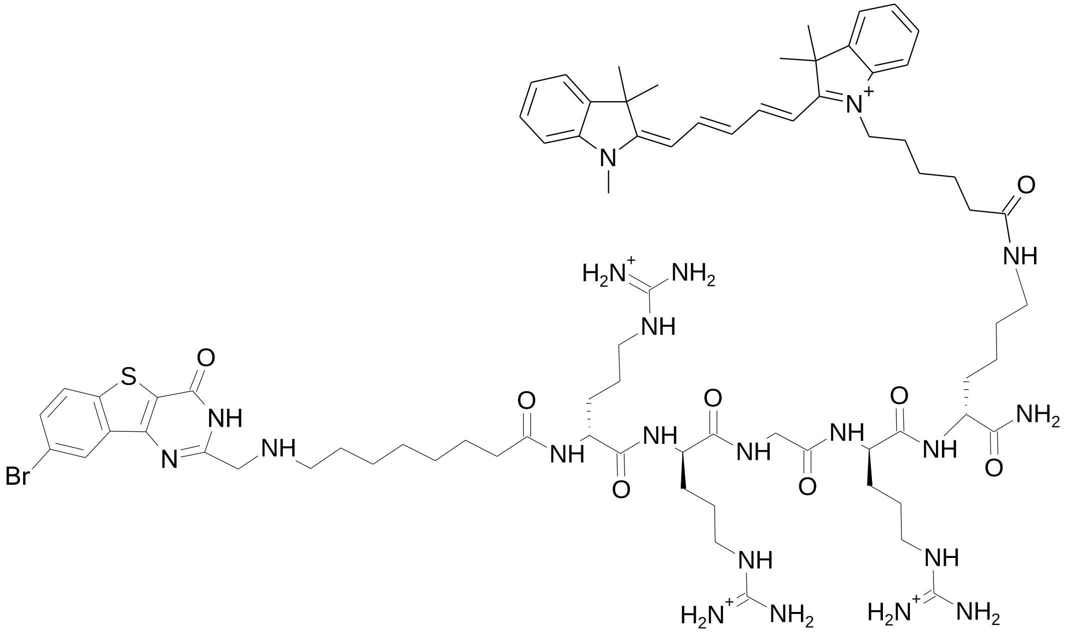<br>$C_{77}H_{111}BrN_{21}O_8S$	1569.78856	1569.77928

7<br>(ARC-2076)	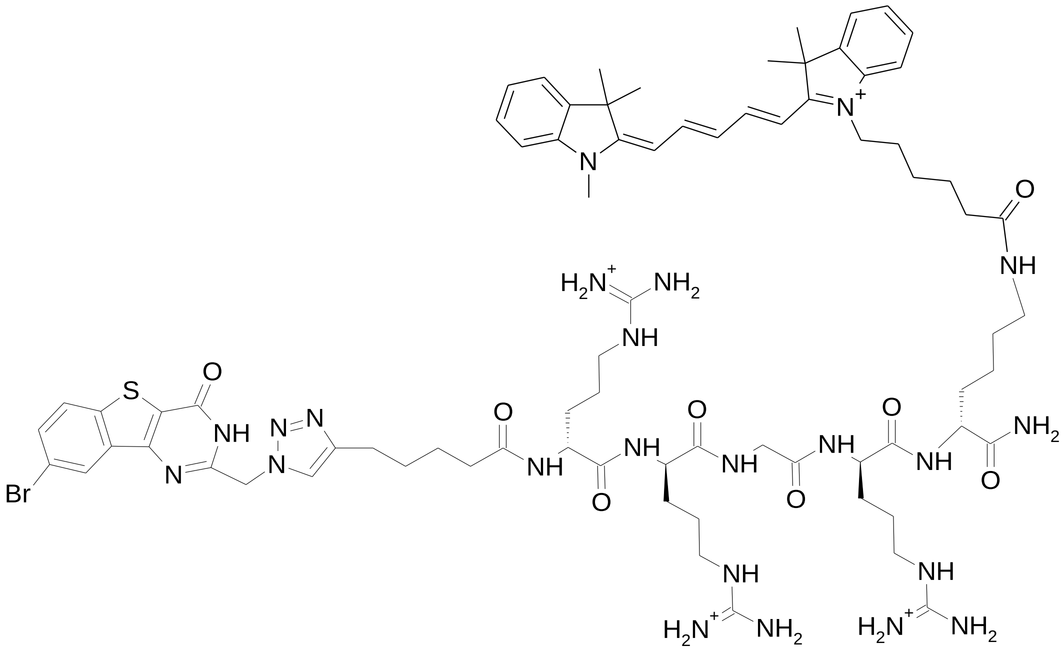<br>$C_{76}H_{105}BrN_{23}O_8S$	1579.74829	1579.73782

8<br>(ARC-2065)	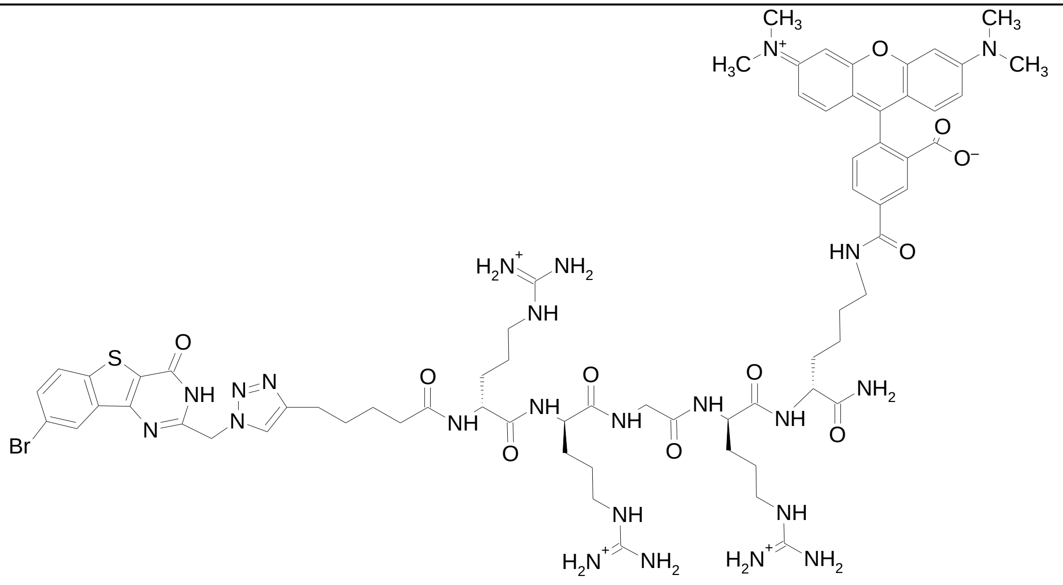<br>$C_{69}H_{88}BrN_{23}O_{11}S$	1526.59483	1526.59999
PIM peptide	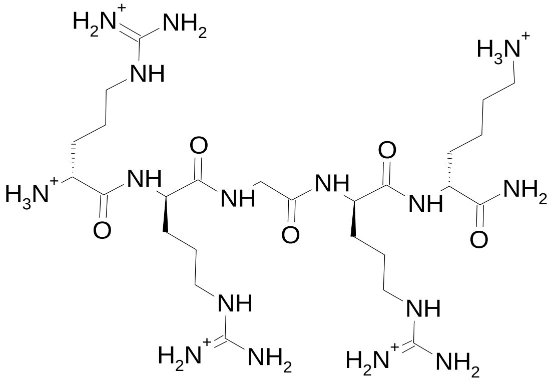<br>$C_{26}H_{54}N_{16}O_5$	670.34668	670.44580

ARC-2073	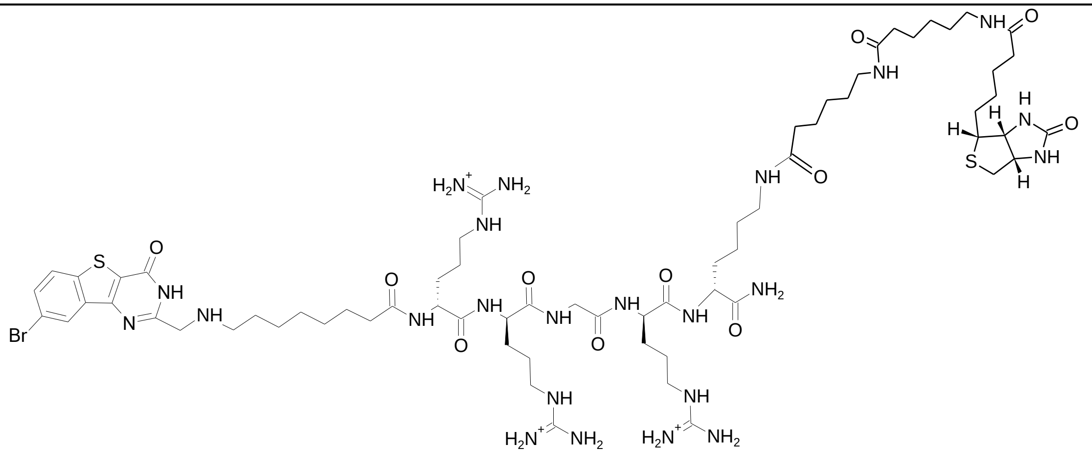<br>$C_{67}H_{110}BrN_{23}O_{11}S_2$	1555.73208	1555.73643
ARC-2090	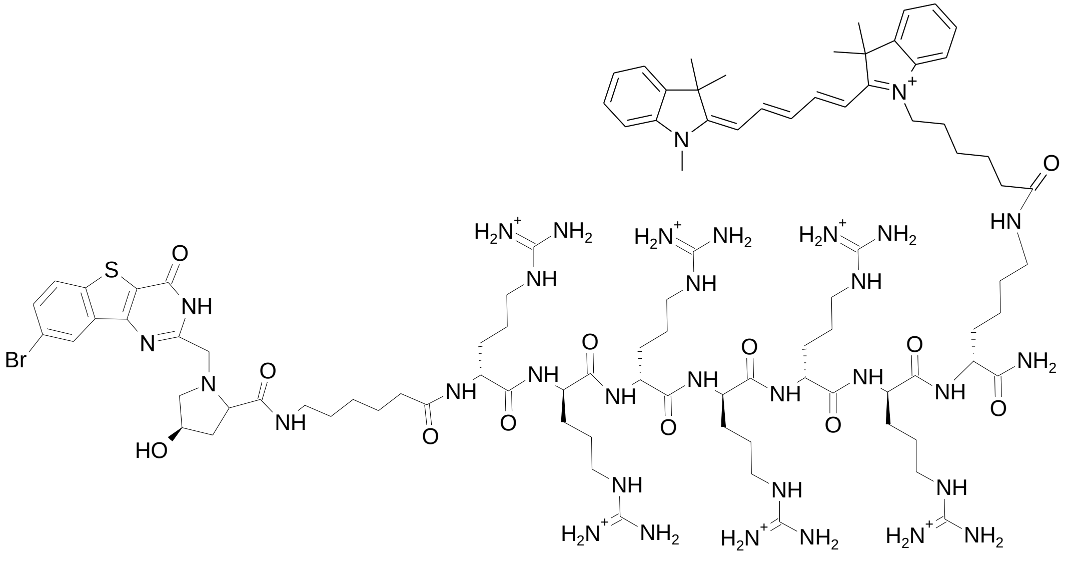<br>$C_{96}H_{147}BrN_{33}O_{12}S$	2066.0818	2066.0805

Table S3. Selectivity profiling of ARC inhibitors (1 μM) towards a panel of PKs

	Residual activity, %				
**PK**	**ARC-3125**	**ARC-3126**	**Cpd 1**	**Cpd 2**	**Cpd 3**
ABL	106 ± 0	116 ± 5	94 ± 8	89 ± 5	91 ± 12
AMPK	6 ± 0	38 ± 4	92 ± 9	91 ± 4	47 ± 6
ASK1	93 ± 0	121 ± 11	99 ± 0	102 ± 5	101 ± 2
Aurora A	84 ± 1	132 ± 8	99 ± 6	106 ± 3	97 ± 3
Aurora B	100 ± 4	114 ± 20	102 ± 19	98 ± 2	111 ± 1
BRK	82 ± 41	81 ± 4	126 ± 8	114 ± 11	114 ± 13
BRSK1	84 ± 2	50 ± 10	107 ± 5	101 ± 0	90 ± 11
BRSK2	107 ± 23	100 ± 9	124 ± 12	108 ± 12	96 ± 8
BTK	31 ± 2	43 ± 2	106 ± 10	99 ± 20	82 ± 1
CAMK1	3 ± 11	55 ± 9	81 ± 18	81 ± 6	49 ± 11
CAMKKb	47 ± 1	104 ± 13	117 ± 1	108 ± 1	85 ± 4
CDK2-Cyclin A	17 ± 1	106 ± 5	106 ± 12	105 ± 9	100 ± 0
CDK9-Cyclin T1	55 ± 1	104 ± 3	110 ± 10	101 ± 20	90 ± 18
CHK1	76 ± 1	100 ± 6	122 ± 17	112 ± 6	104 ± 2
CHK2	116 ± 16	76 ± 15	107 ± 9	103 ± 2	52 ± 10
CK1γ2	45 ± 5	66 ± 10	117 ± 3	116 ± 10	88 ± 1
CK1δ	71 ± 4	99 ± 1	104 ± 4	99 ± 3	88 ± 12
CK2	30 ± 2	8 ± 3	77 ± 9	63 ± 12	57 ± 7
CLK2	-1 ± 5	57 ± 15	97 ± 9	24 ± 2	30 ± 1
CSK	85 ± 2	91 ± 18	141 ± 10	112 ± 20	116 ± 5
DAPK1	18 ± 1	49 ± 18	85 ± 3	60 ± 11	88 ± 3
DDR2	84 ± 13	93 ± 3	104 ± 22	107 ± 7	112 ± 6
DYRK1A	28 ± 7	99 ± 6	74 ± 4	5 ± 2	37 ± 15
DYRK2	54 ± 6	88 ± 6	85 ± 17	64 ± 5	83 ± 22
DYRK3	22 ± 2	82 ± 1	43 ± 6	34 ± 2	55 ± 4
EF2K	103 ± 1	85 ± 10	104 ± 2	98 ± 7	102 ± 3
EIF2AK3	11 ± 11	60 ± 6	94 ± 7	98 ± 5	113 ± 13
EPH-A2	108 ± 2	113 ± 13	116 ± 5	113 ± 10	132 ± 6
EPH-A4	97 ± 4	107 ± 13	97 ± 2	103 ± 16	95 ± 5
EPH-B1	106 ± 12	96 ± 6	103 ± 9	99 ± 15	106 ± 11

EPH-B2	102 ± 8	107 ± 4	95 ± 16	100 ± 5	96 ± 14
EPH-B3	104 ± 3	94 ± 10	107 ± 26	106 ± 2	79 ± 3
EPH-B4	119 ± 7	101 ± 10	104 ± 2	118 ± 11	98 ± 13
ERK1	89 ± 7	84 ± 7	119 ± 16	125 ± 6	107 ± 6
ERK2	161 ± 4	166 ± 1	101 ± 14	102 ± 1	104 ± 5
ERK5	47 ± 1	92 ± 1	111 ± 9	77 ± 5	93 ± 4
ERK8	18 ± 9	49 ± 30	109 ± 8	78 ± 17	83 ± 2
FGF-R1	100 ± 19	102 ± 3	142 ± 8	126 ± 10	110 ± 2
GCK	56 ± 4	88 ± 14	112 ± 0	114 ± 2	97 ± 7
GSK3β	140 ± 13	80 ± 0	110 ± 12	75 ± 8	64 ± 8
HER4	120 ± 9	110 ± 5	111 ± 13	114 ± 15	105 ± 8
HIPK1	61 ± 1	95 ± 15	111 ± 9	60 ± 12	60 ± 3
HIPK2	16 ± 2	75 ± 15	103 ± 13	48 ± 2	50 ± 7
HIPK3	21 ± 0	93 ± 2	116 ± 15	72 ± 12	57 ± 7
IGF-1R	4 ± 0	114 ± 24	77 ± 8	91 ± 6	58 ± 7
IKKβ	80 ± 1	104 ± 2	97 ± 0	100 ± 11	90 ± 6
IKKε	77 ± 4	118 ± 12	116 ± 9	104 ± 15	122 ± 12
IR	22 ± 6	117 ± 6	122 ± 7	139 ± 35	113 ± 23
IRAK1	16 ± 3	120 ± 2	131 ± 17	134 ± 21	113 ± 11
IRAK4	73 ± 2	97 ± 2	110 ± 3	108 ± 8	107 ± 7
IRR	20 ± 34	75 ± 4	99 ± 2	99 ± 5	110 ± 1
JAK2	115 ± 5	116 ± 9	93 ± 14	82 ± 16	72 ± 21
JNK1	82 ± 13	116 ± 4	100 ± 13	112 ± 2	115 ± 13
JNK2	123 ± 7	38 ± 13	107 ± 1	91 ± 7	129 ± 21
JNK3	120 ± 2	121 ± 5	92 ± 0	108 ± 3	113 ± 22
Lck	226 ± 7	132 ± 28	94 ± 18	103 ± 18	78 ± 6
LKB1	49 ± 28	114 ± 11	107 ± 1	118 ± 4	115 ± 13
MAP4K3	17 ± 1	81 ± 7	119 ± 1	118 ± 3	105 ± 2
MAP4K5	102 ± 3	50 ± 12	107 ± 12	112 ± 10	98 ± 3
MAPKAP-K2	51 ± 5	100 ± 3	107 ± 10	107 ± 2	114 ± 4
MAPKAP-K3	48 ± 1	43 ± 7	111 ± 6	110 ± 11	85 ± 5
MARK1	76 ± 4	94 ± 0	110 ± 15	113 ± 1	106 ± 2
MARK2	86 ± 4	103 ± 7	106 ± 10	107 ± 4	79 ± 0

MARK3	72 ± 1	112 ± 17	100 ± 6	102 ± 7	100 ± 8
MARK4	92 ± 3	119 ± 7	124 ± 14	128 ± 3	90 ± 12
MEKK1	77 ± 1	103 ± 2	108 ± 16	119 ± 5	98 ± 2
MELK	34 ± 4	79 ± 1	86 ± 5	75 ± 9	73 ± 11
MINK1	86 ± 1	104 ± 16	129 ± 2	106 ± 13	133 ± 14
MKK1	32 ± 27	100 ± 6	93 ± 4	94 ± 1	92 ± 6
MKK2	68 ± 5	117 ± 14	102 ± 5	108 ± 11	96 ± 7
MKK6	110 ± 10	105 ± 1	122 ± 2	121 ± 3	126 ± 10
MLK1	100 ± 8	122 ± 21	112 ± 13	98 ± 8	101 ± 9
MLK3	98 ± 2	117 ± 10	105 ± 5	107 ± 1	94 ± 2
MNK1	67 ± 4	89 ± 5	102 ± 3	66 ± 7	89 ± 8
MNK2	19 ± 4	84 ± 17	80 ± 3	47 ± 2	96 ± 10
MPSK1	75 ± 3	79 ± 11	113 ± 21	113 ± 15	110 ± 2
MSK1	6 ± 8	29 ± 1	106 ± 13	53 ± 4	90 ± 2
MST2	73 ± 1	106 ± 6	115 ± 11	128 ± 35	84 ± 6
MST3	51 ± 24	102 ± 5	104 ± 3	112 ± 8	98 ± 1
MST4	71 ± 4	125 ± 22	143 ± 10	121 ± 8	95 ± 32
NEK2α	21 ± 3	91 ± 2	108 ± 5	119 ± 12	92 ± 1
NEK6	97 ± 0	85 ± 17	107 ± 11	126 ± 5	117 ± 23
NUAK1	7 ± 15	47 ± 7	95 ± 14	102 ± 14	74 ± 6
OSR1	73 ± 2	116 ± 12	114 ± 7	114 ± 4	107 ± 14
p38α MAPK	53 ± 8	116 ± 4	113 ± 10	116 ± 10	110 ± 7
p38β MAPK	80 ± 6	94 ± 4	108 ± 14	113 ± 4	100 ± 6
p38δ MAPK	70 ± 6	111 ± 4	109 ± 7	117 ± 1	108 ± 8
p38γ MAPK	119 ± 11	129 ± 10	116 ± 6	115 ± 0	111 ± 1
PAK2	29 ± 4	72 ± 5	99 ± 9	100 ± 10	91 ± 4
PAK4	50 ± 2	71 ± 42	91 ± 0	98 ± 14	77 ± 13
PAK5	25 ± 6	89 ± 2	112 ± 3	100 ± 2	92 ± 5
PAK6	34 ± 11	103 ± 5	119 ± 8	110 ± 15	86 ± 8
PDGFRA	97 ± 0	116 ± 3	115 ± 2	101 ± 16	108 ± 4
PDK1	123 ± 7	111 ± 13	116 ± 3	121 ± 7	118 ± 16
PHK	104 ± 11	128 ± 10	109 ± 3	98 ± 4	78 ± 6
PIM1	0 ± 0	0 ± 0	9 ± 1	5 ± 3	5 ± 1

PIM2	0 ± 1	10 ± 11	72 ± 3	7 ± 1	15 ± 1
PIM3	7 ± 1	13 ± 3	17 ± 1	4 ± 1	6 ± 0
PINK	---	---	116 ± 5	121 ± 17	92 ± 0
PKA	7 ± 3	97 ± 0	100 ± 9	85 ± 6	82 ± 10
PKBα	-1 ± 1	80 ± 4	101 ± 6	67 ± 7	72 ± 12
PKBβ	1 ± 1	86 ± 10	115 ± 26	101 ± 5	54 ± 6
PKCα	81 ± 9	115 ± 17	112 ± 2	102 ± 4	110 ± 5
PKCζ	14 ± 1	92 ± 4	95 ± 6	118 ± 11	89 ± 2
PKCγ	86 ± 9	98 ± 8	99 ± 11	97 ± 3	105 ± 9
PKD1	18 ± 4	111 ± 10	128 ± 13	134 ± 14	82 ± 3
PLK1	40 ± 4	50 ± 16	99 ± 1	99 ± 1	97 ± 7
PRAK	112 ± 3	97 ± 7	104 ± 13	89 ± 0	79 ± 7
PRK2	16 ± 6	58 ± 15	92 ± 2	94 ± 12	67 ± 4
RIPK2	37 ± 6	99 ± 6	62 ± 7	72 ± 0	71 ± 8
ROCK 2	5 ± 1	65 ± 17	114 ± 17	61 ± 5	62 ± 5
RSK1	19 ± 1	82 ± 10	95 ± 6	31 ± 4	74 ± 12
RSK2	17 ± 1	86 ± 15	109 ± 11	92 ± 10	106 ± 11
S6K1	13 ± 0	75 ± 2	98 ± 2	85 ± 1	55 ± 6
SGK1	9 ± 5	75 ± 9	110 ± 19	89 ± 3	95 ± 4
SIK2	34 ± 4	109 ± 6	110 ± 5	107 ± 10	68 ± 3
SIK3	89 ± 7	117 ± 15	106 ± 2	97 ± 9	66 ± 6
SmMLCK	66 ± 1	116 ± 39	98 ± 5	98 ± 9	94 ± 0
Src	153 ± 22	103 ± 2	97 ± 3	110 ± 12	119 ± 1
SRPK1	54 ± 1	89 ± 5	102 ± 1	113 ± 7	91 ± 2
STK33	84 ± 20	107 ± 6	110 ± 2	106 ± 3	91 ± 0
SYK	94 ± 10	75 ± 20	133 ± 15	123 ± 3	114 ± 2
TAK1	9 ± 4	88 ± 17	107 ± 3	101 ± 1	99 ± 10
TAO1	32 ± 6	95 ± 3	106 ± 10	49 ± 0	80 ± 4
TBK1	68 ± 3	105 ± 12	113 ± 29	113 ± 10	46 ± 4
TESK1	102 ± 3	99 ± 14	111 ± 11	106 ± 13	105 ± 1
TGFBR1	108 ± 18	109 ± 2	103 ± 8	106 ± 7	95 ± 10
TIE2	104 ± 9	106 ± 0	110 ± 6	109 ± 9	103 ± 12
TLK1	78 ± 1	91 ± 10	118 ± 1	122 ± 9	109 ± 10

TrkA	97 ± 7	112 ± 21	106 ± 17	128 ± 13	95 ± 2
TSSK1	63 ± 6	97 ± 16	98 ± 9	108 ± 0	100 ± 10
TTBK1	123 ± 25	103 ± 4	116 ± 5	97 ± 7	99 ± 6
TTBK2	101 ± 24	77 ± 2	109 ± 6	125 ± 12	105 ± 15
TTK	45 ± 1	110 ± 4	113 ± 0	94 ± 15	86 ± 9
ULK1	89 ± 9	119 ± 6	107 ± 5	99 ± 6	108 ± 11
ULK2	34 ± 3	104 ± 9	115 ± 5	127 ± 9	104 ± 11
VEG-FR	95 ± 41	85 ± 0	110 ± 9	96 ± 3	90 ± 5
WNK1	105 ± 33	107 ± 34	96 ± 4	93 ± 1	104 ± 6
YES1	104 ± 8	98 ± 12	110 ± 0	104 ± 12	124 ± 1
ZAP70	105 ± 8	83 ± 30	95 ± 21	87 ± 7	53 ± 14

Mean values ± standard error are shown (N = 2).

Table S4. HPLC (C18 reversed phase) purity data for the previously unpublished compounds

**Compound**		**Molecular formula**	**HPLC data**			
#	Code		Gradient	t_R/min	Purity (%)	λ_{max}/nm
-	ARC-1451	$C_{89}H_{130}BrN_{33}O_{15}Se$	18 … 42/9.5 min	3.73	100	560
-	ARC-3126	$C_{58}H_{102}BrN_{31}O_9S$	5 … 40/7 min	3.61	98.3	307
**1**	ARC-2067	$C_{44}H_{73}BrN_{20}O_7S$	20 … 95/11 min	7.33	96.1	307
**2**	ARC-2059	$C_{45}H_{74}BrN_{19}O_7S$	20 … 95/11 min	8.63	97.2	342
**3**	ARC-2060	$C_{42}H_{65}BrN_{22}O_7S$	15 … 95/7 min	7.67	97.5	342
**4**	ARC-2061	$C_{44}H_{68}BrN_{21}O_7S$	5 … 40/7 min	3.62	96.1	342
**5**	ARC-2062	$C_{50}H_{81}BrN_{20}O_9S$	5 … 40/7 min	3.75	97.3	342
**6**	ARC-2074	$C_{77}H_{111}BrN_{21}O_8S$	50 … 95/7 min	6.19	97.4	650
**7**	ARC-2076	$C_{76}H_{105}BrN_{23}O_8S$	50 … 95/7 min	6.46	98.4	650
**8**	ARC-2065	$C_{69}H_{88}BrN_{23}O_{11}S$	74 … 82/7 min	5.20	96	560
PIM peptide		$C_{26}H_{54}N_{16}O_5$	5 … 25/8 min	3.61	96	206
ARC-2090		$C_{96}H_{147}BrN_{33}O_{12}S$	25 … 95/7 min	3.38	95	650
ARC-2073		$C_{67}H_{110}BrN_{23}O_{11}S_2$	50 … 95/7 min	3.54	96.7	342

Separation was achieved with a Luna C18 5 μm column (250×4.6 mm Phenomenex) protected by a 5 μm Luna C18 4×2.0 mm guard column. Mobile phase A: 0.1% TFA in water, mobile phase B: 0.1% TFA in ACN and a flow of 1 mL/min were applied. The speed of the gradient is specified in the table.

Supplementary Figures

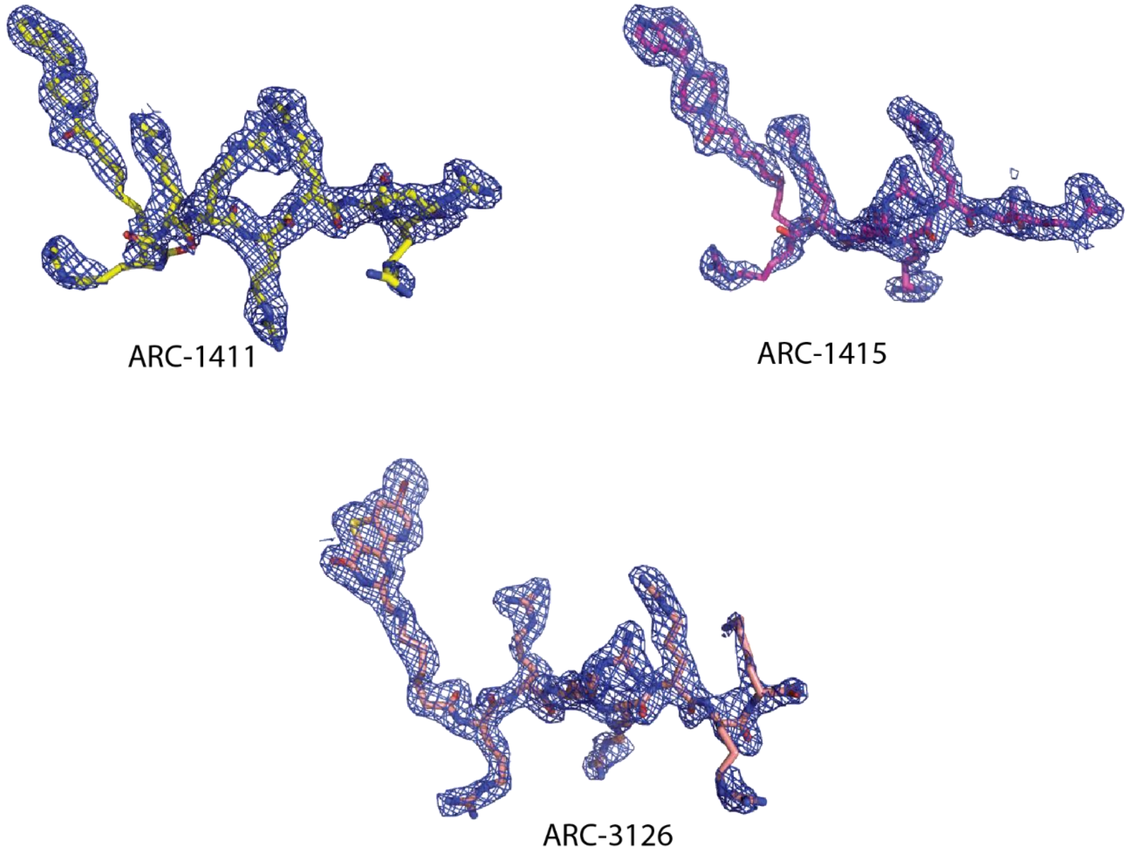

Figure S1. $2F_o$-F_c electron density maps contoured at 1σ for the inhibitors in co-crystal structures with PIM-1. Inhibitors are shown in stick representation.

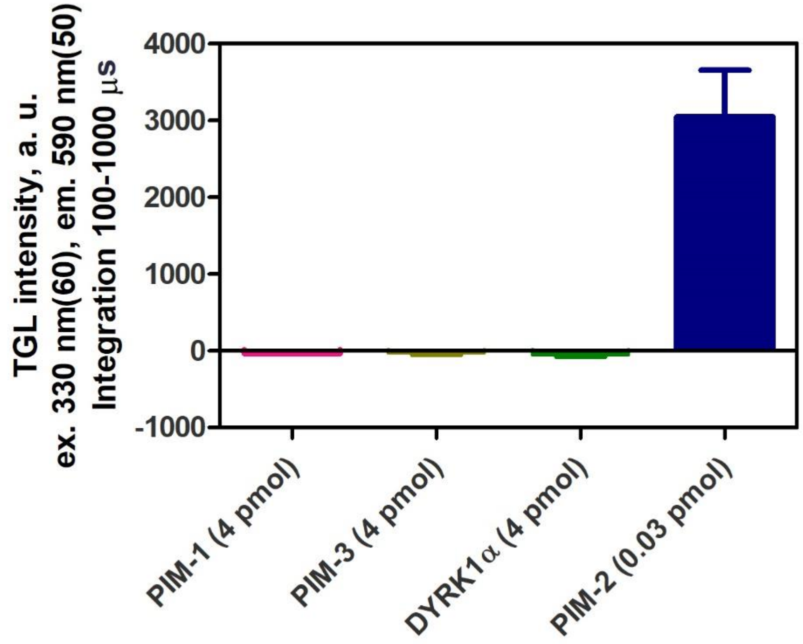

Figure S2. Specificity of D1D2 antibody to PIM-2 detection.

ARC-2073 (4 pmol) was immobilized onto STRA-coated surface, incubated with the kinases, and detected with D1D2 (84 fmol) and G0506 (110 fmol). Mean values ± SEM are shown (N = 2). Of four protein kinases tested, only complex with PIM-2 yielded time-resolved signal reflecting binding of the detection reagent D1D2-G0506 to PIM-2.

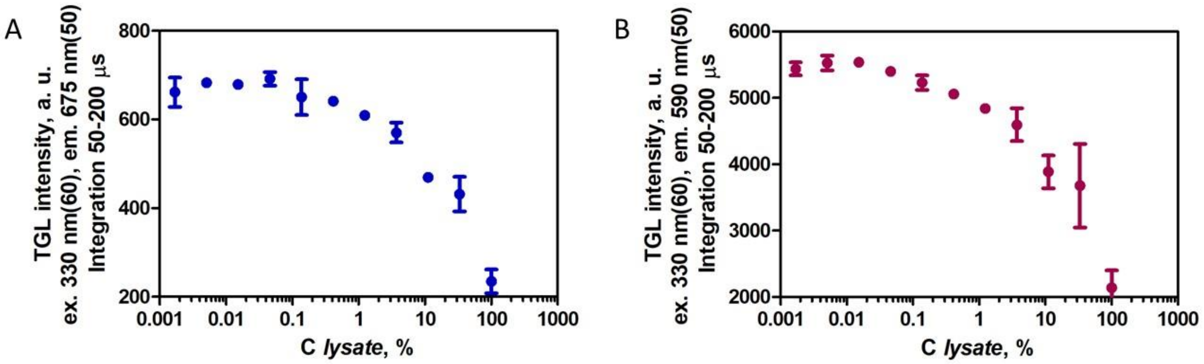

Figure S3. Stability of ARC-1451/PIM-2 complex in PC-3 cell lysate.

The stability of complex in a biological matrix was assessed in solution by FRET measurement from ARC-1451 (6 nM) to D1D2-AF647 (10 nM) in the presence of PIM-2 (10 nM). (A) Signal from FRET acceptor D1D2-AF647; (B) signal from FRET donor ARC-1451/PIM-2. Mean values ± SEM are shown (N = 2). The presence of increased percentage of lysate results in decline of both donor and acceptor signal intensitites due to the matrix effects.

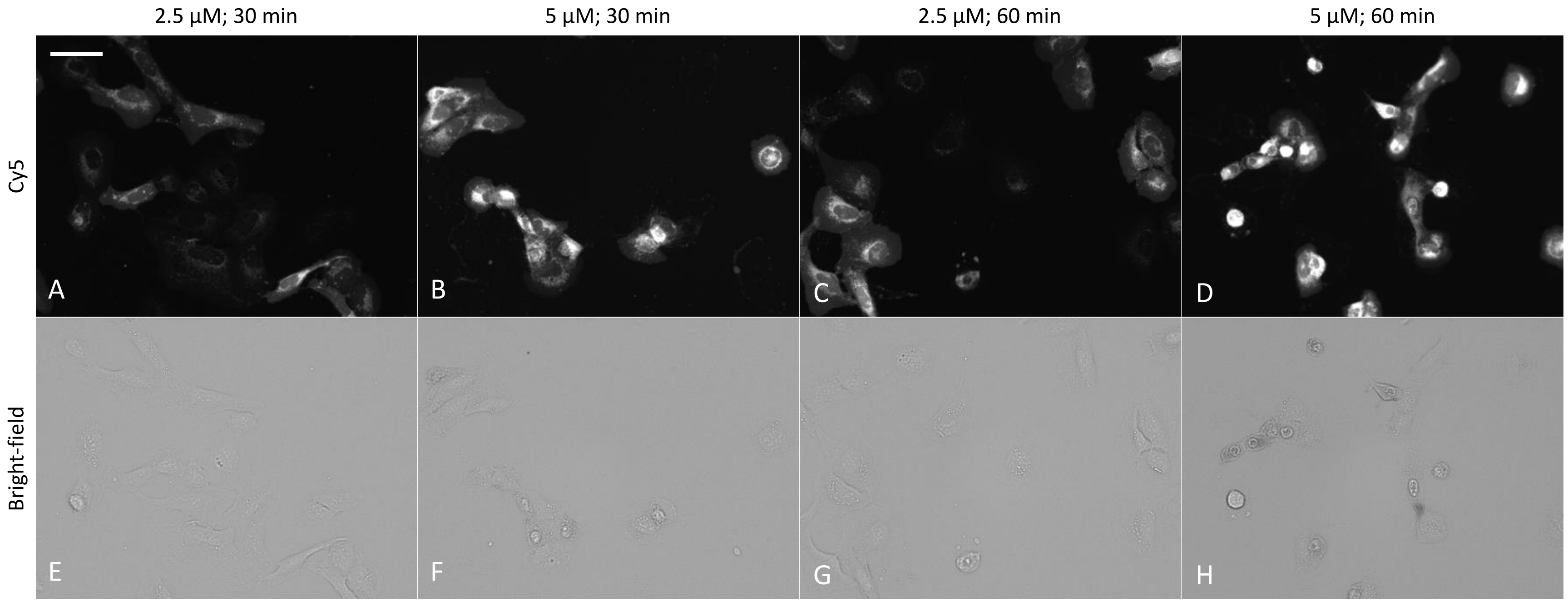

Figure S4. Time- and concentration-dependence of the cellular uptake of compound **6** into live U2OS cells.

(A-D) Cy5 channel; (E-H) bright-field. Incubation conditions are listed above the images. Representative images from a single experiment are shown; in all wells, imaging settings were identical (see Materials and Methods section of the main text). Scale bar: 50 µm. The signal intensity in the fluorescence channel reflects efficient internalization of the probe following incubation with 2.5 µM or higher concentration in the incubation medium. Following 60 min incubation with 5 µM concentration of probe, darker regions are observable in bright-field images resulting from accumulation of the probe in cells which causes absorption of visible light by the Cy5 dye.

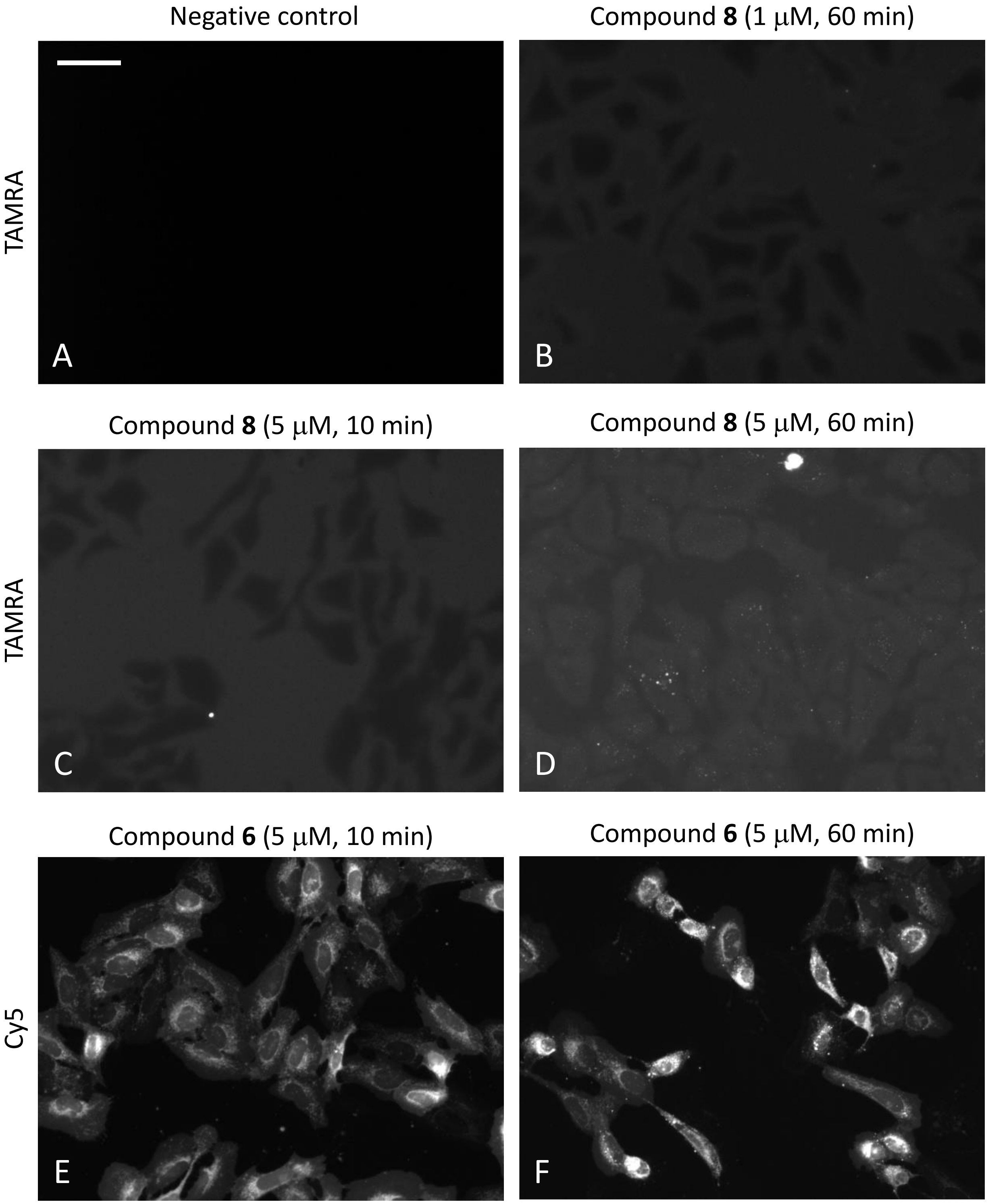

Figure S5. Comparison of cellular uptake of compound **8** (labelled with TAMRA) and compound **6** (labelled with Cy5) into live U2OS cells.

(A) TAMRA signal intensity in the well not treated with ARC probes; (B-D) TAMRA signal intensity in wells incubated with compound **8**; (E-F) Cy5 signal intensity in wells incubated with compound **6**. Incubation conditions are listed above the images. Scale bar: 50 µm. Representative images from a single experiment are shown; for TAMRA channel, more intense imaging settings (LED intensity, integration time, detector gain) were used than for Cy5 channel (see Materials and Methods section of the main text). Very low intracellular signal can be seen for compound **8** (following incubation with 5 µM probe for 60 min), whereas incubation with lower concentration of the probe or shortening of incubation time results in cellular exclusion of the compound. The nature of the fluorescent dye thus impacts the cellular internalization and non-specific binding properties of the conjugate.

ARC-2090
5 µM; 60 min

Compound **6**
5 µM; 60 min

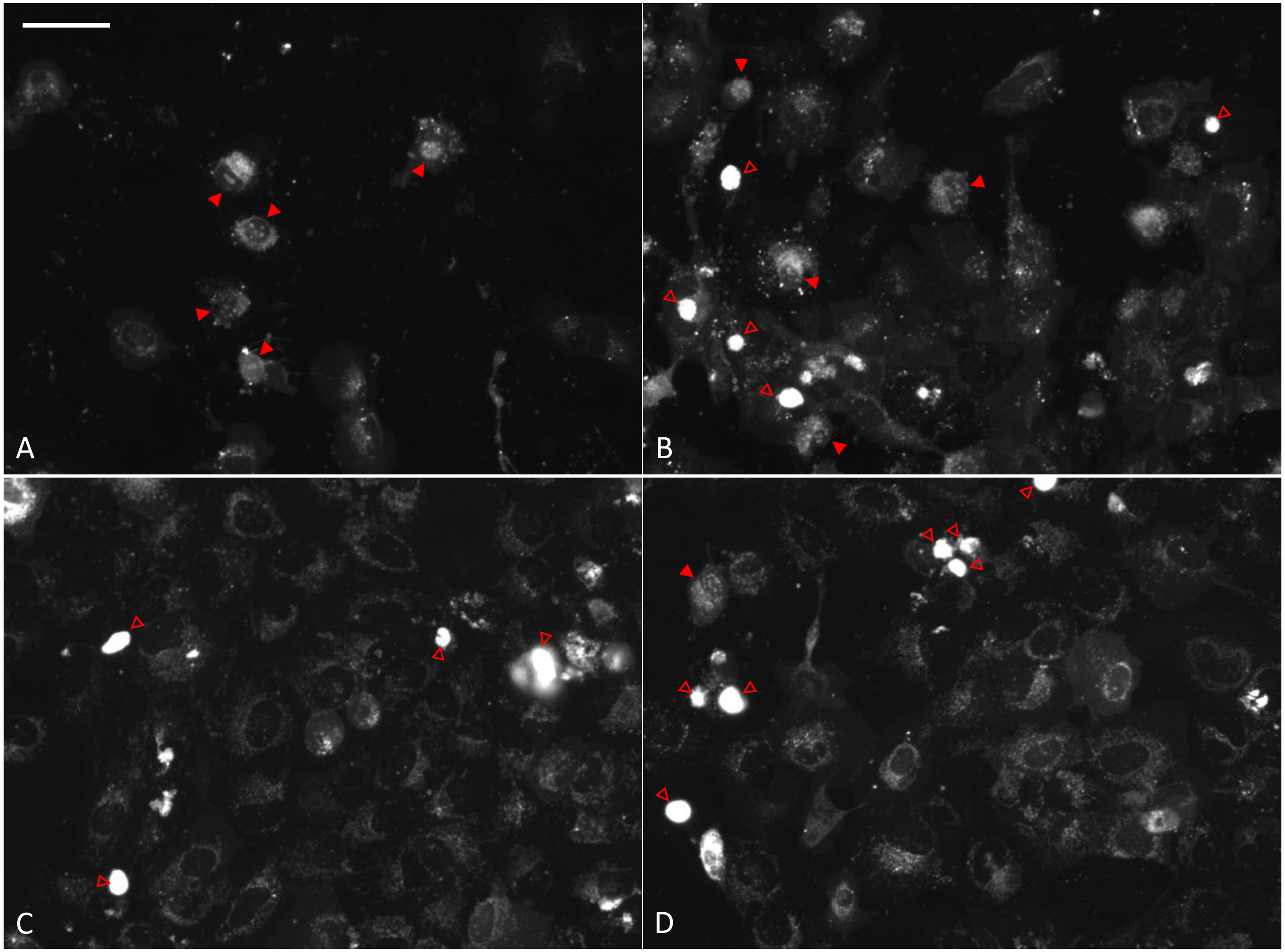

Figure S6. Intracellular localization of compounds labelled with Cy5 and incorporating different number of Arg residues.

Imaging was carried out in live U2OS cells; (A, B) ARC-2090 (6 Arg); (C, D) compound **6** (3 Arg). Incubation conditions are listed on the left; representative images from a single experiment are shown (replicate images for the same treatment condition represent identically treated wells).

Scale bar: 50 μm. Red filled triangles point to the cells featuring strong staining in the nuclear compartment; red empty triangles indicate dead cells. In case of ARC-2090, higher proportion of cells with intense staining of nucleoli is evident, which can be attributed to the incorporation of of (D-Arg)$_6$-peptide in this probe.

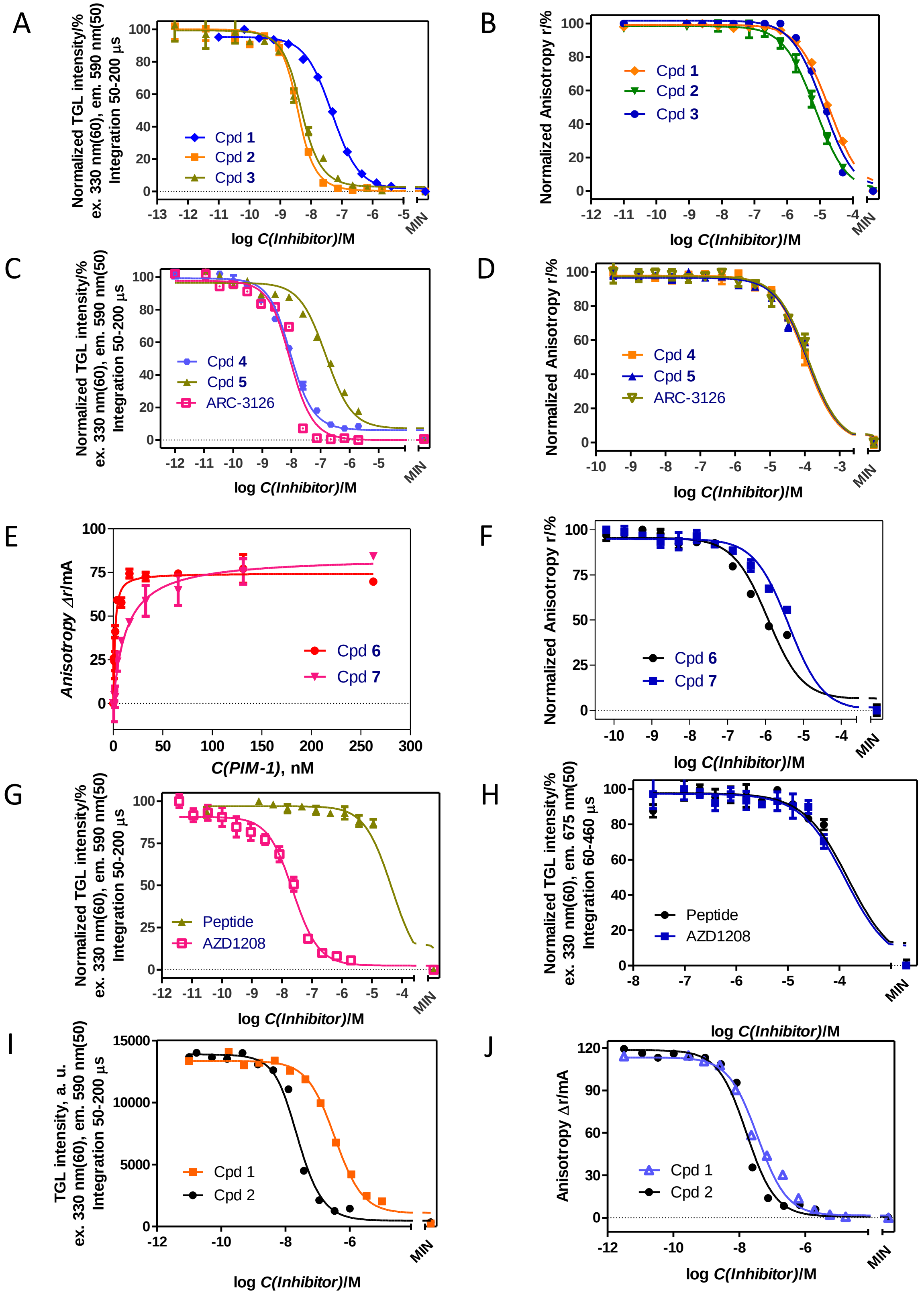

Figure S7. Biochemical characterization of the synthesized compounds and commercially available inhibitors.

(A) Displacement of ARC-1451 (2 nM) from the complex with PIM-1 (3 nM) by compound **1**, **2**, or **3**. (B) Displacement of ARC-583 (2 nM) from the complex with PKAcα (3 nM) by **1**, **2**, or **3**. (C) Displacement of ARC-1451 (2 nM) from the complex with PIM-1 (3 nM) by **4**, **5**, or ARC-3126. (D) Displacement of ARC-583 (0.5 nM) from the complex with PKAcα (2 nM) by **4**, **5**, or ARC-3126. (E) Binding of **6** and **7** (1.5 nM) to PIM-1. (F) Displacement of ARC-583 (0.5 nM) from the complex with PKAcα (2 nM) by **6** and **7**. (G) Displacement of ARC-1451 (2 nM) from the complex with PIM-1 (3 nM) by PIM peptide or AZD1208. (H) Displacement of ARC-1188 (0.5 nM) from the complex with PKAcα (3 nM) by PIM peptide or AZD1208. (I) Displacement of ARC-1451 (0.5 nM) from the complex with PIM-2 (5 nM) by **1** or **2**. (J) Displacement of ARC-1451 (2 nM) from the complex with PIM-3 (5 nM) by **1** or **2**. Mean values ± SEM are shown (N = 2). The adenosine analogue moiety of compound **2** enhances its affinity to PIM-1 and PIM-2 (compared to compound **1**). The structural modifications performed to compound **2** allowed conservation of nanomolar affinity to PIM kinases.

Supplementary Video

Video S1. Colocalization studies of PIM-targeting compound **6** with fluorescently tagged PIM-1 *vs* peroxisomal marker (following 48 h transfection) in live U2OS cells.

The PIM-1-mRFP (right part) and mCherry-Peroxisomes-2 (left part) are shown in green and compound **6** in red on overlayed images; Z-stack is shown as time stack (3 frames per second) for better visualization of the colocalization over the whole cell.
